# Refining acceptance and commitment therapy in daily life (ACT-DL) for clinical care: Optimization and mixed-methods evaluation of usability and implementation feasibility

**DOI:** 10.1016/j.invent.2026.100979

**Published:** 2026-07-22

**Authors:** Lotte Uyttebroek, Joanne R. Beames, Martien Wampers, Evelyne van Aubel, Jeroen Weermeijer, Inez Myin-Germeys

**Affiliations:** a Center for Contextual Psychiatry, Psychiatry Research Group, Department of Neurosciences, KU Leuven, Leuven, Belgium

**Keywords:** Acceptance and commitment therapy, Ecological momentary interventions, Experience sampling methodology, Mixed-methods, Blended care

## Abstract

**Background:**

Blended care can enhance Acceptance and Commitment Therapy (ACT) by combining face-to-face sessions with digital tools. The ACT in Daily Life (ACT-DL) intervention integrates therapy sessions with a smartphone-based Ecological Momentary Intervention to promote psychological flexibility and to transfer ACT into daily life.

**Aim:**

This study describes an iterative optimization and evaluation of ACT-DL, in terms of feasibility (i.e., practical implementation), usability (i.e., ease of using ACT-DL), and integration into clinical practice by clinicians.

**Methods:**

ACT-DL was optimized using qualitative interviews with clients and resulted in adding a third component: a clinician dashboard to review app data. In a second step, we evaluated ACT-DL 2.0 in an uncontrolled pre-post implementation pilot study with 7 clinicians and 30 clients. Feasibility was assessed using indicators including adoption, attrition, engagement, attendance and adaptability. Usability data were gathered via weekly and post-intervention questionnaires. Integration in care was explored through thematic template analysis of clinician interviews.

**Results:**

ACT-DL was feasible, with clients attending on average 6.75 (*SD* = 7.83) sessions and engaging with 37.3% (*SD* = 27.16) of the programmed Ecological Momentary Assessment (EMA) questionnaires. Clients and clinicians found several elements usable (e.g. exercises, EMA items), though advanced features such as visualizing data remained challenging. Dashboard complexity, lack of time, limited mastery and client characteristics were barriers to integrating ACT-DL into practice.

**Conclusion:**

ACT-DL is feasible, however mixed usability results highlight the need for further optimization and implementation strategies to support clinicians in fully integrating ACT-DL across clinical care contexts.

## Highlights

• ACT-DL is feasible for clients in terms of attendance and engagement
• Core ACT-DL components, including therapeutic exercises, ESM items and visualizations, were perceived as relevant by clinicians and their clients; however, overall system usability remains challenging
• ESM supports symptom monitoring, treatment evaluation and decision-making
• Sufficient and adequate clinician training is essential for successful implementation
• Participatory research is necessary for optimizing ESM-based interventions

## 1 Introduction

Blended care is a promising approach to amplify psychological therapy by integrating face-to-face therapy with digital mental health interventions (Erbe et al., 2017). Literature has demonstrated the potential for integrating ambulatory methods, such as Ecological Momentary Interventions (EMI) (Heron and Smyth, 2010) and Ecological Momentary Assessment (EMA) (Stone and Shiffman, 1994), into the treatment of mental health disorders (Balaskas et al., 2021; Bell et al., 2017). EMIs use smartphone technology to deliver interventions in daily life, such as reminders and therapeutic exercises, essentially offering a 24/7 available “therapist in your pocket”. The use of EMIs in addition to face-to-face therapy facilitates the application of learned skills beyond the therapy room, fostering their integration and generalization into daily life (Heron and Smyth, 2010; Myin-Germeys et al., 2016). The delivered interventions can be tailored based on EMA data, which is collected through repeated self-report questionnaires assessing mood, behaviours, activities and symptoms as they occur in daily life, with minimal recall biases and high ecological validity (Dao et al., 2021; Myin-Germeys et al., 2018; Myin-Germeys and Kuppens, 2021). EMA/EMI in addition to therapy can increase client empowerment, self-awareness and self-management (Bos et al., 2020; Myin-Germeys et al., 2024; Simons et al., 2015; Folkersma et al., 2021; Bonnier et al., 2025) offering a real-world focused and person-centred approach to psychotherapy.

Acceptance and Commitment Therapy in Daily Life (ACT-DL) is an evidence-based blended care intervention that combines protocolized face-to-face therapy sessions with a smartphone application, the ACT-DL app, which uses an EMA-guided EMI to deliver exercises and increase emotional awareness in daily life (Myin-Germeys et al., 2022; Reininghaus et al., 2019; Vaessen et al., 2019). ACT-DL employs cognitive and behavioral techniques to promote psychological flexibility, defined as the ability to act toward or in accordance with personal values and goals, regardless of unpleasant experiences or sensations (Hayes, 2016; Hayes et al., 2006). Psychological flexibility is achieved through six skills or ACT processes (acceptance, cognitive defusion, self-as-context, contact with the present moment, values and committed action), which in turn, contribute to better well-being (Hayes, 2016; Hayes et al., 2006). In ACT-DL, the ACT processes are introduced in the therapy sessions and are practised in daily life with the app, creating a powerful approach to bridge the therapy room and everyday life.

From an mHealth research lifecycle perspective, ACT-DL has progressed through multiple iterations of feasibility testing, user-evaluation and clinical trials, with each step informing subsequent optimization (Wilson et al., 2018). First, ACT-DL was examined in a small inpatient study assessing feasibility and preliminary effects (Batink et al., 2016), followed by two randomized controlled trials (RCT): one using group ACT-DL in individuals with emerging depressive and psychotic symptoms (van Aubel et al., 2020) and another using individual ACT in early psychosis (Myin-Germeys et al., 2022). ACT-DL led to a decrease in researcher-rated depressive symptoms (van Aubel et al., 2020) and improvements in negative symptoms and functioning (Myin-Germeys et al., 2022). The second RCT (INTERACT) also included an early feasibility check with 16 participants (Vaessen et al., 2019), broader feasibility and acceptability assessments (van Aubel et al., 2024) and qualitative interviews (Bouws et al., 2025). These studies demonstrate feasibility in terms of engagement and acceptability (Myin-Germeys et al., 2022; Vaessen et al., 2019; Batink et al., 2016; van Aubel et al., 2020; van Aubel et al., 2024), but also highlight barriers such as EMA burden and difficulties integrating ACT-DL in daily life, particularly during work, school or social activities (van Aubel et al., 2020; van Aubel et al., 2024; Bouws et al., 2025). Understanding such barriers is important for guiding further iterations of ACT-DL that better match client needs and enhance its clinical impact.

Since ACT-DL is a blended care intervention, each optimization requires a precise consideration of how it could practically be used in real-world therapeutic contexts (e.g. for different purposes, within different health care settings) (Greenhalgh and Abimbola, 2019; Greenhalgh et al., 2017). Clinicians and clients must learn to navigate ACT-DL and translate the EMA data into a coherent and meaningful narrative for discussion. User-centered design (Morton et al., 2020) can help translate real-world needs and preferences into actionable changes that enhance the relevance, acceptance and implementation of interventions (Veldmeijer et al., 2023). In the current study, we applied user-centered design principles to optimize the existing version of ACT-DL based directly on user feedback. Because additional refinements may influence how the intervention functions in practice, it is important to evaluate the effects of these changes before moving toward broader implementation or scale-up.

Implementation pilot studies allow the evaluation and refinement of interventions and develop implementation strategies before conducting an RCT or hybrid implementation study (Pearson et al., 2020). Evaluating implementation outcomes such as feasibility and usability is crucial for further adoption (Venkatesh et al., 2012; Proctor et al., 2011), since digital EMA tools should support healthcare without creating excessive demands for clients and clinicians (Piot et al., 2022; Bos et al., 2019). Feasibility refers to whether ACT-DL can be successfully used and integrated in care delivery, often operationalized as recruitment rate, intervention uptake, and retention (Proctor et al., 2011). Usability refers to all pragmatic aspects of interacting with ACT-DL in the context of clinical care, including ease of use, learnability, efficiency, satisfaction, and technical and practical problems (ISO, 2019; Weermeijer et al., 2023; Nielsen, 2021). Understanding real-world feasibility and usability of an optimized version of ACT-DL will offer valuable insight for its practical application and will facilitate understanding of what is needed to implement ACT-DL more widely in different health care contexts.

### 1.1 Current study

This study presents a new iteration of ACT-DL that was optimized by incorporating end-user experiences into the design. We demonstrate the optimization process and assess the optimized version in an implementation pilot study. Through optimizing ACT-DL, we added a new component: a clinician dashboard. Since this was not part of previous iterations, we aim (1) to assess the feasibility, usability and usefulness of the optimized ACT-DL for clinicians and clients, (2) to evaluate clinicians' perceived competencies in utilizing ACT-DL in the sessions, and (3) to explore how clinicians utilized and integrated ACT-DL into their care setting. Given that research on the implementation of EMA/EMI in clinical settings is still an emerging area and the nature of this pilot study, all analyses are exploratory.

### 1.2 Transparency and reporting

This study was post-registered at the Open Science Framework (OSF) on 16th October 2024 (https://osf.io/pjbew/?view_only=02e4ff2468ea491cab327394b752fabe). All study materials are accessible on the OSF project page. Minor deviations to the registration plan, decision rules for data cleaning, and code are also documented on the OSF (https://osf.io/dch7r/?view_only=14cfc716ff594c7ca5e96ce3666d574e).

## 2 Methods

### 2.1 Optimization of ACT-DL

Findings from self-report and qualitative client interviews from the INTERACT study (Reininghaus et al., 2019) informed the optimization of ACT-DL (van Aubel et al., 2024; Bouws et al., 2025). In the qualitative interviews, clients reported challenges in integrating the ACT-DL app into daily life, due to the fixed questionnaire schedule and the use of a study phone. Clients suggested extending the app's availability beyond the intervention, emphasizing ACT exercises and metaphors, and receiving feedback on app activities (Bouws et al., 2025). These highlighted the need for flexibility, personalization and the integration of feedback of app-use in the session. The optimized ACT-DL included 1) eight written protocolized face-to-face therapy sessions, described in the manual with a clear link to the daily life app, 2) the app for practice in between sessions, and 3) the dashboard, which were both built into m-Path's EMA software platform (https://m-path.io/landing/). The different components are described in more detail below. Table 1 presents an overview of the changes that were made from ACT-DL 1.0 to ACT-DL 2.0.

**Table 1:** Overview of the changes from ACT-DL 1.0 to ACT-DL 2.0.

ACT-DL 1.0	ACT-DL 2.0
Fixed EMA protocol	Flexible EMA protocol: clinicians could adjust session order or repeat sessions (starting with problem exploration and creative hopelessness and ending with psychological flexibility was recommended)
Fixed assessment schedule: 8 questionnaires per day for 3 consecutive days after the therapy session	Flexible and adaptable assessment schedule: the number of daily questionnaires and assessment days could be personalized by the clinician and further adjusted throughout the intervention period
Fixed set of items in the EMA questionnaires	Personalization of items in the EMA questionnaires: clinicians could select additional EMA items from a list or create new items (see OSF for all EMA items)
Random exercise or metaphor at the end of the EMA questionnaires	Tailored exercises, with computational algorithm based on ACT skill with lowest momentary score
No feedback	Feedback on app use presented on the ACT-DL dashboard using graphic visualizations for discussion in the sessions
Study device	Personal device

#### 2.1.1 ACT-DL app

**Fig. 1:**
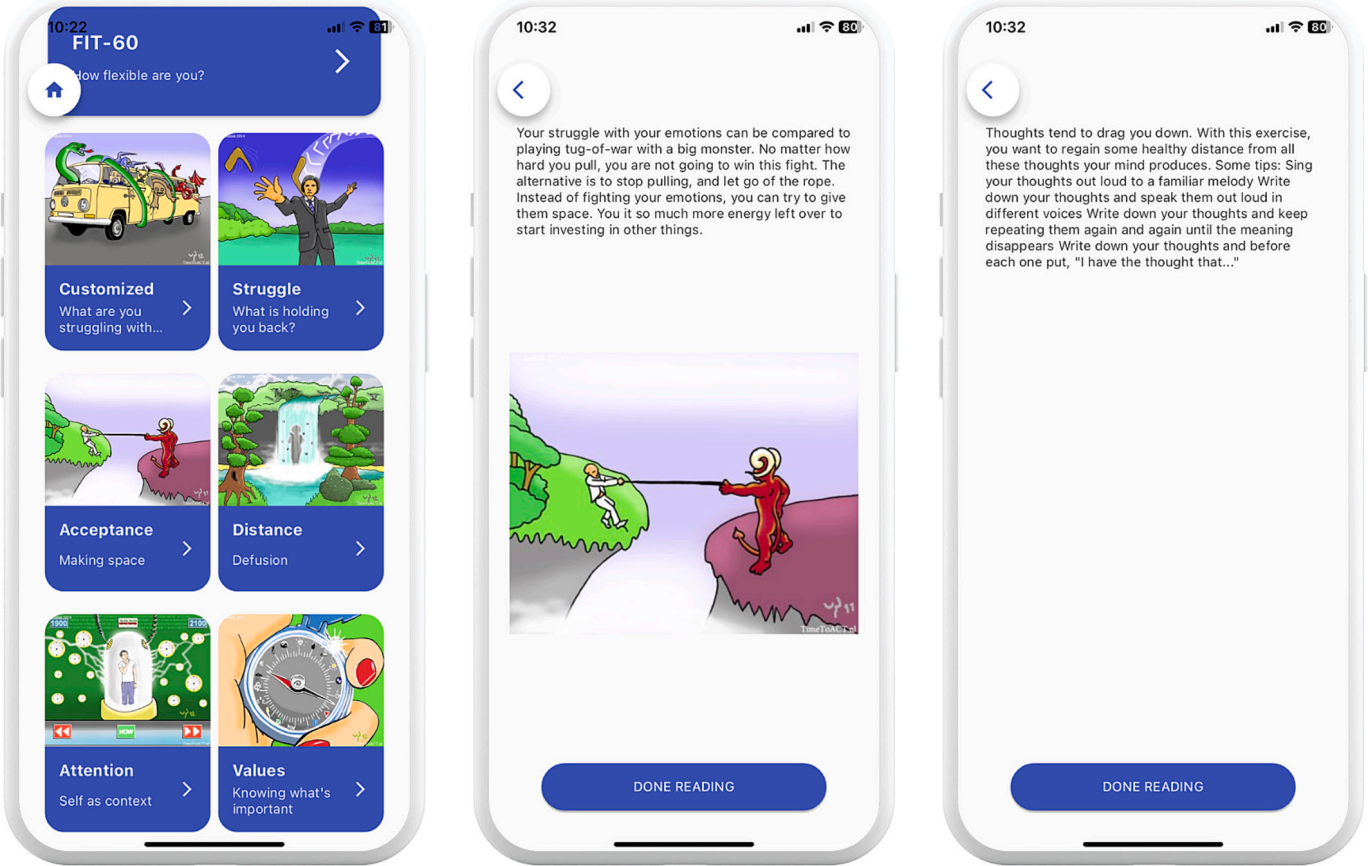
ACT-DL app home screen and example exercises*.*

The ACT-DL app included EMA questionnaires and ACT exercises. The EMA questionnaires assessed momentary mood, context, activities, ACT skills (e.g., “*I'm struggling with my thoughts and emotions*” – acceptance) and optionally, symptoms (since symptom reduction is not the primary goal of ACT). Completing an EMA questionnaire took approximately 2 min. Clients could also initiate a morning and evening questionnaire using a button on the home screen at time of waking up and/or going to bed. The morning questionnaire assessed sleep quality, while the evening questionnaire assessed significant positive and negative events in the past day, session specific questions (e.g., *“Today I had difficult experiences, thoughts, or bodily sensations”* - acceptance) and app feasibility. In the app, ACT materials were organized into tiles corresponding to each ACT-skill and included exercises (visual, textual and audio), psycho-educational videos and metaphors (Fig. 1). Exercises in the app were either EMA-initiated or user-initiated. First, the EMA-initiated exercises were offered after each EMA questionnaire. A computational algorithm identified the ACT skill with the lowest momentary score (based on 6 ACT items) and presented a corresponding exercise. For example, if a client scored lowest on acceptance, an exercise targeting acceptance was offered. Second, clients could independently navigate through the app and self-select exercises by choosing a specific ACT skill tile (user-initiated). Lastly, if clients wished to engage in an exercise but were unsure which one to choose, they could access the customized tile (Fig. 1). In this case, the same computational algorithm used in the EMA-initiated pathway selected an exercise tailored to the client's current ACT skills.

#### 2.1.2 ACT-DL dashboard

**Fig. 2:**
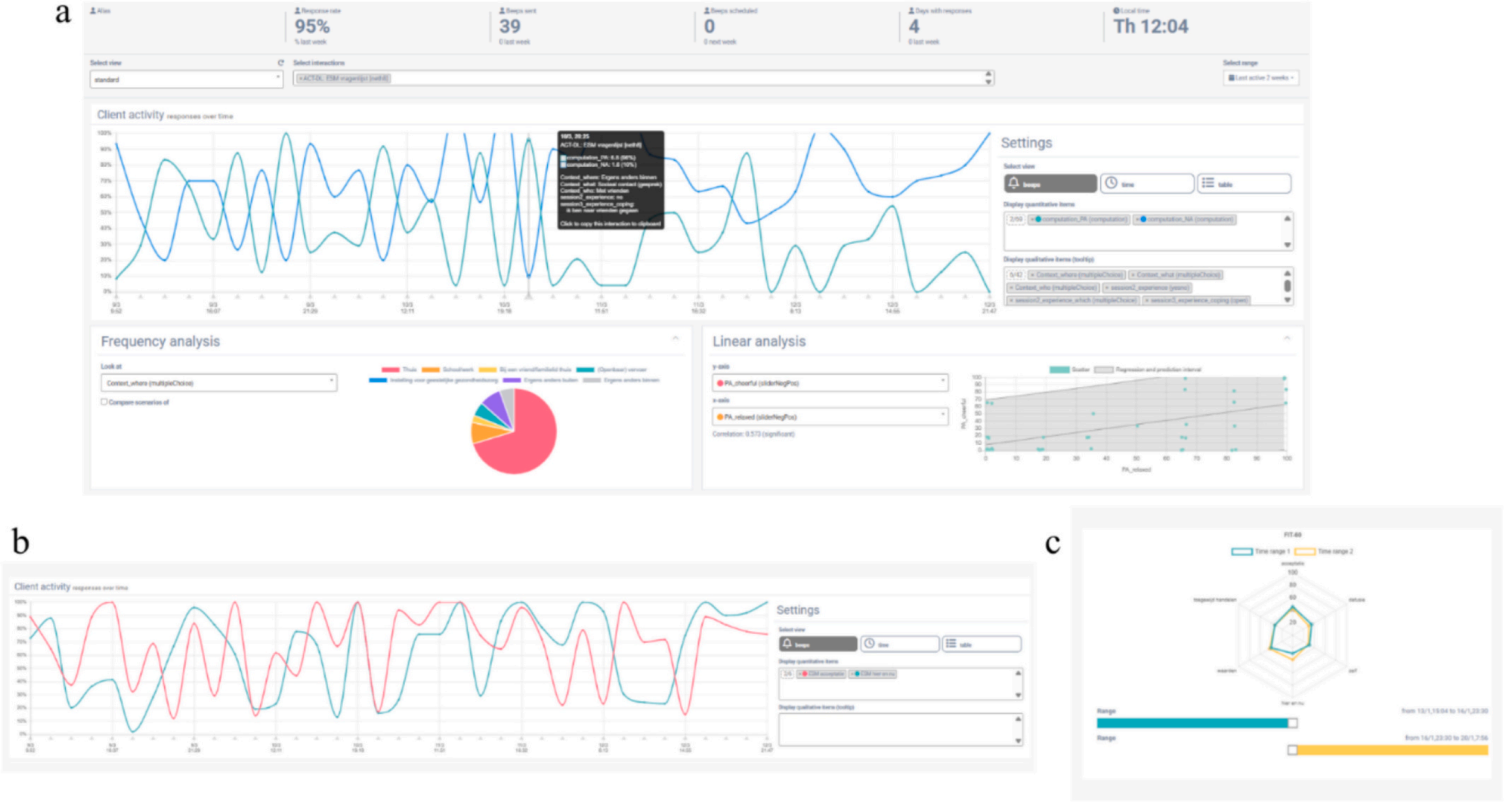
ACT-DL dashboard for clinicians. *Note.* a = home page of the clinician dashboard showing key EMA compliance metrics (e.g., percentage of completed responses in recent weeks, number of active days), time-series plots of affect, and frequency summaries of contextual variables, b = beep-level time-series displays of two ACT skills (e.g., acceptance, present-moment awareness), c = visualization of psychological flexibility across different time periods.

Clinicians utilized the dashboard to set up and personalize the EMA protocol by manually scheduling the number of EMA questionnaires sent and the EMA items from a list (see OSF for all items). The default contained 20 items. At a later stage, clinicians reviewed and discussed the app data on the dashboard (Fig. 2) using a set of customized visualizations. These included contextual information (e.g., pie chart of location or social context), fluctuations over time (e.g. positive and negative affect, symptoms), in which it was possible to zoom into specific data points to relate other information to this data point (e.g. location, company). Additionally, the dashboard included ACT-specific visualizations (e.g. time series of ACT skills, completion and responses of exercises, psychological flexibility).

### 2.2 Evaluation of ACT-DL

#### 2.2.1 Design and participants

This mixed-methods, observational implementation pilot study used an uncontrolled pre-post design to assess feasibility, usability and integration of the optimized ACT-DL. We aimed to recruit 12 practicing ACT clinicians, each enrolling a minimum of four clients. In accordance with the CONSORT guidelines for pilot studies (Eldridge et al., 2016), the sample size was based on practical considerations, given the early stage of ACT adoption in Belgium.

Broad inclusion criteria were used to reflect real-world mental health care. Clinicians had to 1) be a licensed psychologist or psychiatrist, 2) have completed a 5-day foundational ACT training course, 3) have at least one year of ACT experience, 4) sufficiently speak Dutch and 5) have access to a computer. Clients had to 1) be 16 or older, 2) seek help for mental health complaints, regardless of diagnosis, 3) sufficiently speak Dutch and 4) have a smartphone with mobile internet. Clients following concurrent protocolized therapy (such as Cognitive Behavioral Therapy) were excluded.

#### 2.2.2 Ethical considerations

This study was approved by the Medical Ethical Committee of UZ Leuven (S64619). All participants were informed about the study by the research team and were asked to provide written informed consent. For clients younger than 18 years, at least one parent was asked to provide additional informed consent. To reflect real-world circumstances, there was no renumeration for the completion of the EMA questionnaires. Clients received a 10 euros voucher after completion of the study.

#### 2.2.3 Procedures

Between April 2021 and February 2022, we conducted recruitment and data collection remotely due to COVID-19. We contacted clinicians in Flanders (Belgium) via email or telephone through our professional network, healthcare organizations, psychiatric hospitals, and online platforms (www.vindeentherapeut.be and www.vindeenpycholoog.be). These platforms are accessible to help-seeking individuals and provide a comprehensive listing of outpatient psychologists and therapists (e.g. private practices) across all regions of Flanders, including information on their theoretical orientation and contact details. After providing written consent, clinicians completed a set of enrollment questionnaires in REDCap (Research Electronic Data Capture) (Harris et al., 2009) and received the intervention materials and a one-hour online training covering the protocol, dashboard use and visualizations. Clinicians recruited clients from their mental health services and referred them to the research team for written informed consent and pre-intervention questionnaires. Together, they started the intervention in individual or group sessions. At this stage, the app was installed and the intervention was personalized (e.g., number of questionnaires, time interval of beeps, availability of exercises in app). The clients received EMA questionnaires between sessions and completed exercises on the app, which were displayed on the dashboard for discussion. The cycle continued for eight sessions over weekly or bi-weekly appointments. During the intervention period, clinicians also reported technical issues, dashboard usability and perceived competencies on the weeks they used ACT-DL with their clients. After the intervention, all participants completed post-intervention questionnaires and a qualitative interview. Interviews were conducted via Skype, and once the COVID-restrictions were lifted, in person. Interview duration varied between 44 min and 68 min (*M* = 52.42, *SD* = 9.08).

#### 2.2.4 Measures

##### 2.2.4.1 Sociodemographic information

Clinicians provided age, gender, profession and years of experience in ACT. Clients provided age, gender, education level, employment status, medication use, mental health diagnosis and prior experience with therapy. Clients' education was categorized into ‘lower education’, including elementary and secondary education, and ‘higher education’, including tertiary education.

##### 2.2.4.2 Feasibility of ACT-DL

Intervention feasibility was determined based on five indicators (Table 2): adoption, attrition, attendance, engagement and adaptability (Proctor et al., 2011; Gadke et al., 2021; Teresi et al., 2022; Zhong et al., 2023). Based on previous research in individuals with early psychosis (van Aubel et al., 2024), we developed a priori criteria to determine client feasibility based on attendance and engagement. The criteria include: 1) attending a minimum of six therapy sessions, and 2) achieving a minimum engagement of 25% of the scheduled EMA questionnaires. ACT-DL was considered feasible in terms of attendance and engagement if these criteria were met.

**Table 2:** Feasibility indicators for clinicians and clients*.*

Indicator	Definition	Operationalization
Adoption	The intention to try or employ ACT-DL in practice (Proctor et al., 2011)	**Clinicians.** Of the informed clinicians, the number and percentage who were included in the study.
		**Clients.** Of the informed clients, the number and percentage who were included in the study
Attrition^a^	Drop out	**Clinicians**. The number and percentage of clinicians that dropped out during the intervention period.
		**Clients**. The number and percentage of clients that dropped out during the intervention period.
Attendance	Attendance of the face-to-face sessions	Clinician. N/A
		**Clients.** The mean number of completed face-to-face sessions, based on the clinician item at post-intervention *‘How many ACT sessions has your client completed?’*
Engagement^b^	The extent to which clinicians and clients used ACT-DL, including the app and dashboard	**Clinicians.** The mean number of weeks in which the clinician used ACT-DL in the clinical sessions, across all clients, operationalized using the weekly item *‘Have you used the ACT-DL app with one or more of your clients?’*
		**Clients**. Duration of engagement was calculated based on the first questionnaire sent and the last questionnaire answered. Total engagement and with subcomponents^a^ (EMA questionnaires, EMA-initiated exercises, user-initiated exercises, customized user-initiated exercises, morning and evening questionnaires) is calculated using the mean of weekly completed interactions across clients.
Adaptability	The flexibility in the intervention procedure to accommodate client's needs (Gadke et al., 2021)	**Clinicians.** The number and percentage of clients for whom clinicians made changes, based on the item at post-intervention *‘Have you made changes to the ACT-DL protocol?’* and ‘*Have you made changes to the order of the sessions?’*
		Client. N/A

*Note*.

a Client attrition refers to clinician-reported drop-out during the intervention period (e.g., clients discontinuing ACT-DL, therapy, or being discharged). Drop-out during the intervention does not necessarily imply missing post-intervention questionnaires.

b Engagement for the separate interactions (i.e. EMA questionnaires, app) was calculated based on the number of participants with at least one datapoint for that specific interaction.

##### 2.2.4.3 Usability of ACT-DL

Usability was assessed using two adapted versions of the Mhealth App Usability Questionnaire (MAUQ) (Zhou et al., 2019) aiming to evaluate key usability aspects such as ease of use (e.g. *“From the start, I found the app easy to use”*), system interface *(“The dashboard had all the features I expected”*), and usefulness (*“I found the visualizations on the dashboard an added value to the therapeutic process”*). A similar adaptation was previously used to assess the usability of an EMA platform (Weermeijer et al., 2023). Clinicians evaluated the usability of the dashboard using a 19-item version, while clients evaluated the usability of the app using a 21-item version. All items were rated on a 7-point Likert scale, ranging from 1 (strongly disagree) to 7 (strongly agree, with 4 neither disagree, nor agree). Higer scores indicated higher usability. The internal consistency was excellent (Cronbach's alpha of 0.94 for clients and 0.91 for clinicians).

Clinicians reported technical and practical problems with the dashboard weekly using six yes/no items with branched open text items if a problem was reported (e.g., *“Did you experience technical issues with receiving the data”*, and, if yes *“Which technical problem?”*). Clients also reported technical and practical problems regarding the ACT-DL app in the evening questionnaire with the item *“Were there any technical or practical problems with the app today?”* (and, if yes, *“Which technical problem?”*, with multiple choice options).

Perceived usefulness of the exercises in the app was assessed after each completed exercise with a bespoke item *“How useful did you find this exercise?”* on a 7-point Likert scale from 1 (not at all) to 7 (very much).

##### 2.2.4.4 Perceived competencies

Clinicians' perceived competencies in utilizing the dashboard were assessed weekly using four bespoke self-report items: “How competent did you feel … 1) visualizing the data, 2) explaining the data, 3) creating questionnaires and 4) scheduling questionnaires”, on a scale from 1 (not at all) to 7 (very much). If low competencies were indicated, elaborating using open text was stimulated.

##### 2.2.4.5 Qualitative interview

Clinicians were invited to participate in a semi-structured interview after the intervention to gain in-depth understanding of how they used ACT-DL in their sessions. The interview guide covered the EMA items, sampling scheme, data visualizations, the ACT exercises, personalization, and integration and added value of ACT-DL. Given the focus on integration into clinical care, client interviews fall beyond the scope of this study and are not included in the analysis.

#### 2.2.5 Data analysis

Descriptive quantitative analyses were conducted in R (Team RC, 2021) version 4.2.3 to summarize sample characteristics and examine feasibility and usability. To assess client engagement, we used the period of active engagement that fell within the intervention period. We created heatmaps to explore distributions of responses on the individual items of the usability questionnaire. In the heatmap analysis, we identified items with predominantly positive (≥50% of responses ranging from 5 = ‘agree’ to 7 = ‘strongly agree’) and mixed ratings. See the OSF for the R code (https://osf.io/dch7r/?view_only=14cfc716ff594c7ca5e96ce3666d574e).

All clinician interviews were conducted in Dutch and manually transcribed verbatim. The transcripts were pseudonymized by replacing identifiable information with generic descriptors or study IDs (e.g. [HOSPITAL_02] and [CLINICIAN_01]) and analyzed using Nvivo version 15 (Lumivero, 2023) using thematic template analysis (Brooks et al., 2015). Template analysis is a form of thematic analysis (Braun and Clarke, 2021) characterized by hierarchical coding and the development of a coding template that can be iteratively refined. It balances a structure (through a coding template) with flexibility, allowing the template to be adjusted in response to the data, typically combining inductive and deductive coding. In multiple stages, we developed and refined a coding-template using a priori codes based on Daniëls et al. (2023), which proposed a step-by-step approach on how ESM can be integrated into therapeutic sessions. The initial template combined top-down (i.e. based on the a priori codes) and bottom-up codes based on two interviews, which was then iteratively refined and applied to all transcripts by LU (Brooks et al., 2015).

To ensure trustworthiness (Ahmed, 2024), we ensured credibility through prolonged engagement with the data, with authors involved in the study management and data collection. We further strengthened credibility by triangulating the qualitative interview data with the quantitative findings. Detailed reporting on sampling procedures aided transferability. We maintained transparency through thorough documentation of the coding process and regular peer reviews between LU and JRB (Ahmed, 2024).

## 3 Results

### 3.1 Sample characteristics

A total of 7 psychologists (all women) used ACT-DL with clients from their mental health care practice, of which 3 (42.86%) in inpatient care and 4 (57.14%) in outpatient care. They had a mean age of 39.14 years (*SD* = 11.46) and on average 5.86 (*SD* = 4.88) years of experience in ACT therapy. A total of 30 clients received ACT-DL (13 women and 17 males) with a mean age of 32.47 years old (*SD* = 13.56). See Table S1 in the Supplementary Materials for sociodemographic characteristics.

### 3.2 Feasibility of ACT-DL

Table 3 presents feasibility indicators. We approached 159 clinicians, of whom 13 showed interest and received additional information. Ten provided informed consent and 7 used ACT-DL, resulting in a 53.85% adoption rate (7/13). One clinician withdrew, yielding an attrition rate of 14.29% (1/7). Clinicians reported engaging with ACT-DL on average for 10.17 weeks (*SD* = 5.78, range 3–18) and adapted the ACT-DL protocol in 41.7% (*n* = 10) of the cases. Adaptations included shortening sessions, spending several sessions on one skill or incorporating non-ACT sessions. Furthermore, clinicians preferred to adapt the structure and sequencing of ACT-DL sessions to fit their existing therapy sessions with clients (*n* = 18, 75%) rather than following the suggested ACT-DL protocol.

**Table 3:** Feasibility indicators for clinicians and clients.

Indicator	Clinicians	Clients
Adoption, % (n/N)	53.85 (7/13)	88.24 (30/34)
Attrition, % (n/N)	14.29 (1/7)	40 (12/30)
Attendance, M (SD, range)^a^		6.75 (3.91, 1–12)
Engagement, M (SD, range)b,^c^		
Dashboard	10.17 (5.78, 3–18)	
Duration of app engagement		8.83 (7.73, 1–27)
Total app engagement		22.02 (17.29, 0–88)
EMA questionnaires engagement		11.32 (9.74, 0–44)
EMA questionnaires engagement (%)^d^		35.30% (27.68, 0–97.14%)
EMA-initiated exercises		3.58 (4.50, 0–32)
User-initiated exercises		1.05 (2.69, 0–14)
Customized user-initiated exercises		0.20 (0.51, 0–2)
Morning questionnaire		3.56 (2.71, 0–8)
Evening questionnaire		2.96 (2.59, 0–7)
Adaptability^a^, % (n/N)		
Changes in ACT-DL protocol (yes), % (n/N)	41.17 (10/24)	
Changes in order (yes), % (n/N)	75 (18/24)	

a Due to missing data from clinicians regarding intervention completion, ‘client attendance’ and ‘adaptability’ were calculated based on a subsample of 24 participants. For ‘adaptability’, clinicians reported changes made to the ACT-DL protocol for all of their clients separately.

b Clinician engagement is based on n = 6, because one clinician dropped out after the first week of ACT-DL.

c Total app engagement was available for all N = 30 clients and is based on the duration of active use of the ACT-DL app. For engagement with the subcomponents, we included all participants with at least one data point per interaction, yielding sample sizes of n = 29 for the EMA questionnaires, n = 28 for EMA-initiated exercises, n = 18 for the user-initiated exercises, n = 13 for the customized user-initiated exercises, n = 28 for the morning questionnaire, and n = 29 for the evening questionnaire. Engagement results reflect the weekly engagement with the app across participants, across all treatment weeks.

d Mean proportion of engagement was calculated based on the percentage of completed questionnaires per week relative to the total questionnaires received (because the number of questionnaires varied among participants). We then averaged the weekly engagement rates across all participants. On average, clients received 5.21 (*SD* = 1.82, range 3–10) EMA questionnaires per day.

**Fig. 3:**
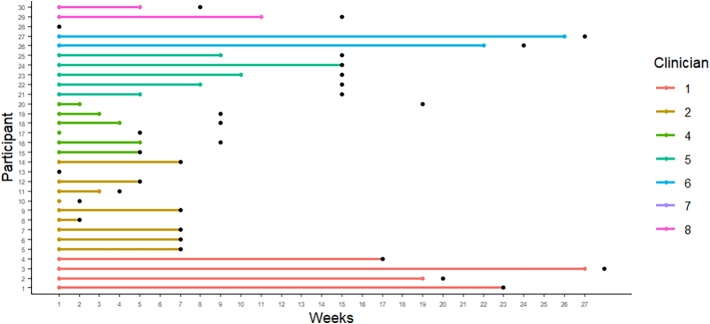
Duration of active engagement with the ACT-DL app per client and clinician. *Note.* The black circles mark the cut-off date used to determine the period of study participation. End-dates were established using a decision hierarchy: 1) the end-date reported by the clinician, 2) drop-out information provided by research team and 3) the date of the post-intervention assessment questionnaires. Any data points recorded after this time were excluded from the analysis.

Clinicians approached 53 clients of whom 34 received additional information, 31 agreed to participate and 30 used ACT-DL, resulting in an adoption rate of 88.24% (30/34). Twelve clients dropped out during the intervention, yielding an attrition rate of 40%. Clients attended on average 6.75 (*SD* = 3.91, range 1–12) sessions and engaged with the app for an average of 8.83 weeks (*SD* = 7.83, range 1–27; see Fig. 3). On a weekly basis, clients used the app on average 22.02 times (*SD* = 17.29, range 0–88), in which they responded to approximately 35.30% (*SD* = 27.68%) of the programmed questionnaires (range 0–97.14%). Engagement with the app varied among clients (Table 3). While almost all (*n* = 29) clients completed at least one EMA questionnaire, only a subset (*n* = 18, 60%) completed a user-initiated exercise. Based on our predefined ‘attendance’ and ‘engagement’ criteria, ACT-DL was deemed feasible.

### 3.3 Usability of ACT-DL

**Fig. 4:**
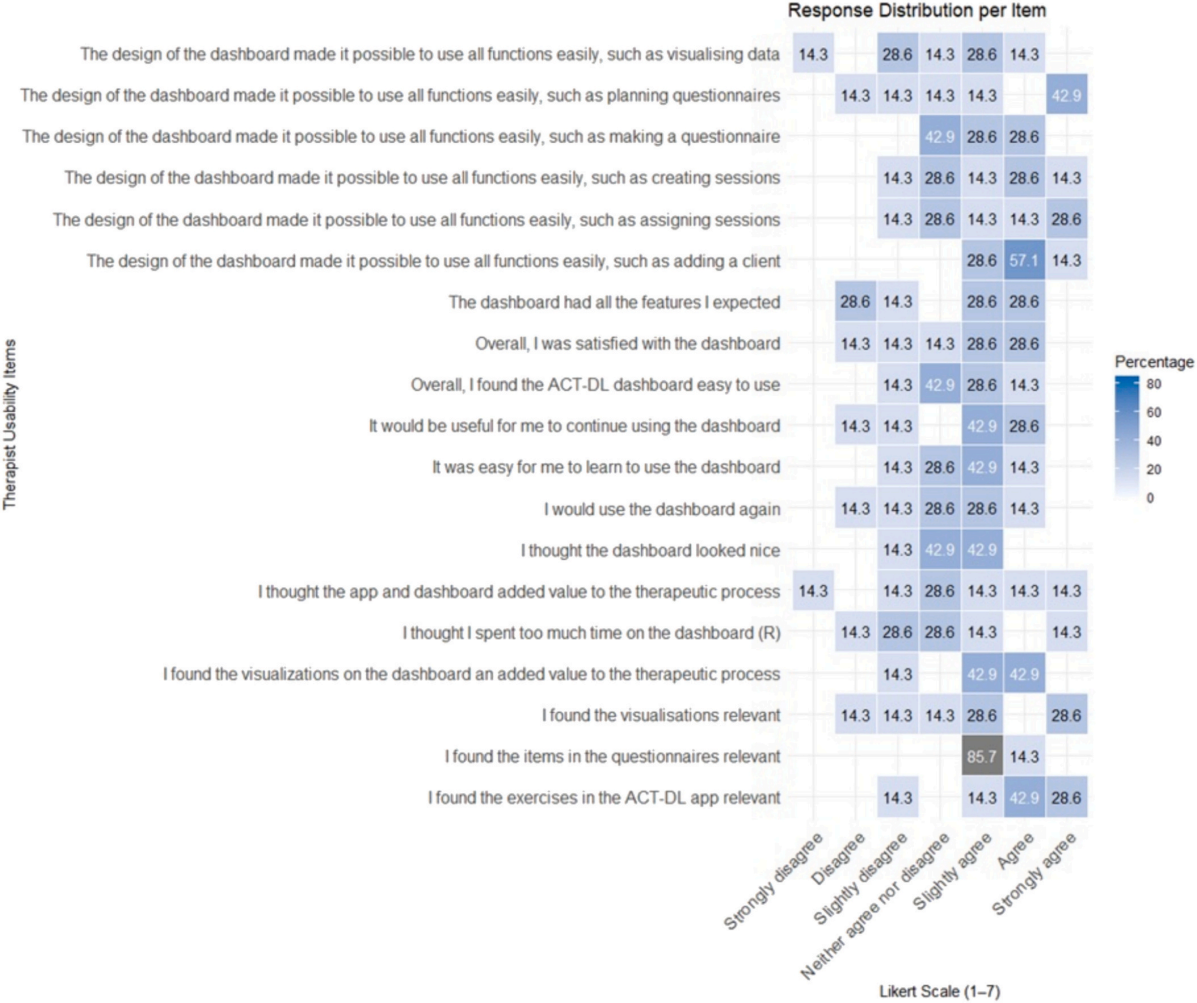
Clinician usability.

Mean clinician responses (N = 7) to the individual items ranged from 3.86 (*SD* = 1.68) to 5.86 (*SD* = 0.69, Supplementary Materials Table S2). The majority of clinicians agreed or strongly agreed that the dashboard was easy to use for assigning sessions and with the relevance of the exercises in the ACT-DL app (Fig. 4). Clinicians generally reported positive usability regarding the dashboard's ease of use to make and plan questionnaires, the relevance of the items and the visualizations, as well as their added value for therapy. Opinions were mixed regarding the dashboard's overall ease of use, interface and time spent on the dashboard. Visualizations were not perceived as usable.

**Fig. 5:**
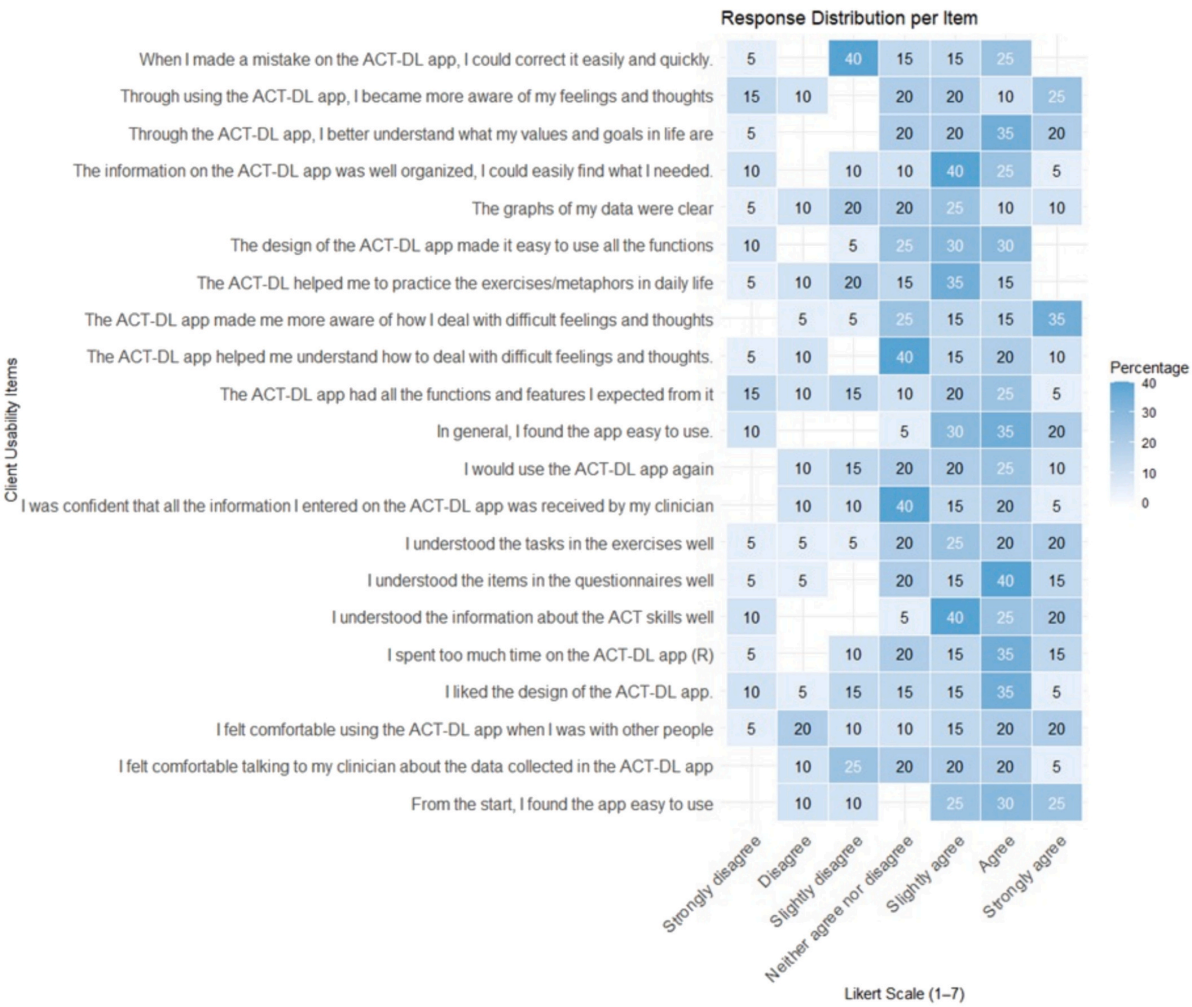
Client usability.

For clients (N = 20), mean individual items scores ranged from 3.9 (*SD* = 1.48) to 5.35 (*SD* = 1.57, Supplementary Materials Table S3). The majority (>50%) of the clients agreed or strongly agreed with the statement on the app's ease of use, on the clarity of the EMA items and with the item *“I spent too much time on the ACT-DL app*” (Fig. 5). Most clients found the app helpful in understanding their values and goals and increasing awareness of how they manage difficult thoughts and emotions. Clients had mixed opinions about correcting mistakes in the app, feeling comfortable discussing the graphs with their clinician, ensuring their input was received by the clinician, as well as the overall clarity of the graphs.

Clients rated usefulness of the EMA-initiated exercises at an average of 4.47 (*SD* = 1.57), the user-initiated exercises at 4.94 (*SD* = 1.54) and the customized user-initiated exercises at 4.10 (*SD* = 1.45) on a 7-point Likert scale.

Clinicians completed 61 weekly questionnaires throughout the intervention period; they reported experiencing problems 23% of the time (14/61), which were mostly about receiving data. Clients completed 734 feasibility items in the app throughout the intervention period; they reported technical problems 7.73% of the time, which were mostly about bugs in the evening questionnaire. See Supplementary Materials Table S4 for more details.

### 3.4 Perceived competencies

Clinicians reported higher mean competencies in constructing (*M* = 5.48, *SD* = 1.22) and planning questionnaires (*M* = 5.41, *SD* = 1.48) compared to visualizing (*M* = 4.54, *SD* = 1.18) and explaining (*M* = 4.48, *SD* = 1.19) the data. The competencies increase over time, with variability across clinicians and within their own engagement. See the Additional Analyses section in the Supplementary Materials for a more in-depth examination (Figs. S1–S4). However, given the exploratory nature, small sample and number of observations, these results should be interpreted with caution.

### 3.5 Integration of ACT-DL into routine care

In the qualitative analysis, we identified four themes including ‘Deciding to use ACT-DL’, ‘ACT-DL Sessions’, ‘ACT-DL EMI’ and ‘Barriers to Integrating ACT-DL’, each with corresponding sub-themes. A summary of the themes, sub-themes, and quotes is provided in Supplementary Materials Tables S5-S8.

#### 3.5.1 Deciding to use ACT-DL

Most clinicians adopted ACT-DL to provide between-session support for clients with access to centralized ACT information. Two clinicians joined the study as part of their search for evidence-based practices.

#### 3.5.2 ACT-DL sessions

##### 3.5.2.1 Group format

Four clinicians delivered group ACT-DL, with three based in inpatient care. Most offered optional individual follow-up sessions to discuss the EMI data more in-depth, rather than discussing the EMI results in the group session. However, one introduced a separate group session to discuss the data, using printed visualizations to support discussion. Some clinicians also noted the role of the group dynamics in how ACT-DL was used. For instance, the printed graphs stimulated curiosity and shared exploration among clients. However, some challenges were also noted when clients had limited data to discuss due to low app engagement or when clients within the group wanted to drop-out. In one group, the clinician decided to not discuss the data, as not all clients had used the app.

##### 3.5.2.2 Individual format

Individual ACT-DL was provided by four clinicians, with three working in outpatient care. Some clinicians reported being careful of balancing the app use with the therapeutic alliance. For example, they minimized app use in the session and adjusted the EMA protocol after the session because they were mindful that clients were paying for the session. Clinicians also reported difficulty finding time outside sessions to learn the dashboard, as this had to be done unpaid.

#### 3.5.3 ACT-DL EMI

##### 3.5.3.1 Personalization

Personalization reflects if and how clinicians aligned the intervention to their clients. All clinicians personalized the EMA protocol (e.g., number of questionnaires per day), with some aligning it with client's daily routines (e.g., school break, therapy schedule). During the intervention, clinicians further adjusted the EMA protocol, such as extending the time clients could respond to the questionnaires or changing the number of questionnaires. One clinician added a custom response option to capture a specific context relevant to the client.

For the group format, clinicians primarily selected items based on group characteristics (e.g., clients with psychosis, general items with a mixed group). Notably, no clinician personalized items at the individual level due to time constraints or risk of overwhelming. Some clinicians also suggested limiting items to essential information, prioritizing ‘need to know’ over ‘nice to know’.

##### 3.5.3.2 Clinician as facilitator

Clinicians facilitated app engagement by reminding clients to complete exercises and EMA questionnaires. While doing so, one noted the challenge of motivating a client without causing pressure. Clinicians also talked with clients about the experiences with EMA, validating and acknowledging emotions such as frustration or discomfort evoked by the questionnaires. Some helped clarify app content by answering questions about exercises or items in the app (e.g., when EMA-initiated exercises addressed topics not covered in the session).

##### 3.5.3.3 Discussion of EMI

This theme reflects whether and how clinicians discussed the app with their clients. For the exercises, one clinician reported actively discussing them in the session; two others addressed the exercises and/or paper homework to some extent, while three utilized their own exercises. Most clinicians reviewed the visualizations but without substantial discussion. One clinician reported insufficient data, two reported a lack of confidence, and another clinician worked with a mixed-user group who were not all participating in the study. Visualizations were used to explore daily life experiences (e.g., sleep), evaluate therapy (e.g., psychological flexibility), or inform treatment-decisions. For example, one clinician concluded therapy after observing positive functioning in daily life.

#### 3.5.4 Barriers to integrating ACT-DL

##### 3.5.4.1 Time

Most clinicians experienced limited time to familiarize with the manual and dashboard. One outpatient clinician expressed having to learn ACT-DL in her free time. Some lacked time to interpret the visualizations, affecting the use of the visualizations.

##### 3.5.4.2 Mastery

Clinicians emphasized the importance of gaining experience with ACT-DL to motivate clients and interpret data effectively. Most expressed the need for in-person training including a “use-case” to build confidence and hands-on experience with the dashboard. Over time, they reported increased comfort in using ACT-DL, for instance by integrating elements in different orders or by omitting less relevant components from the ACT-DL protocol to better fit their therapeutic style, client needs and session format.

##### 3.5.4.3 Usability

Usability hindered use of ACT-DL. Clinicians experienced challenges with navigating the amount of information displayed on the dashboard and with adjusting the questionnaire schedule. Some were unsure whether certain issues were due to user error or missing data. For example, one clinician repeatedly did not receive data because questionnaires were not scheduled for enough days.

##### 3.5.4.4 Client characteristics

Engagement varied according to client characteristics. While some clients were reluctant to participate (e.g., fear or distrust of technology), others (e.g. psychosis or autism spectrum disorder) found it challenging to respond to frequent ESM prompts. For others, however, the app sparked curiosity.

##### 3.5.4.5 Clinical approach

Clinical approach refers to a clinician's therapeutic style or orientation that influenced adoption. Two clinicians felt EMA conflicted with their approach or ACT principles, resulting in limited discussion of the visualizations, and, in one case, dropout. For example, one clinician thought that self-monitoring at fixed times conflicted with the ACT principle of fostering psychological flexibility. Other clinicians perceived the ACT-DL structure as more suitable for group sessions.

## 4 Discussion

### 4.1 Principal findings

This study describes an iteration of optimization and mixed-methods pilot implementation evaluation of a blended care ACT-intervention. ACT-DL was integrated into various health care contexts in Flanders. Some core elements were reported as usable and useful by clients and clinicians, however overall system usability and advanced features such as data visualizations required additional time and effort to be utilized, and several implementation barriers were identified. Overall, this study contributes to the growing literature supporting the feasibility of ACT-DL across diverse clinical populations (Vaessen et al., 2019; Batink et al., 2016; van Aubel et al., 2020; van Aubel et al., 2024).

### 4.2 Feasibility and use of ACT-DL

According to the predefined feasibility criteria based on previous ACT-DL studies (van Aubel et al., 2024), ACT-DL was feasible. Client engagement with the EMA questionnaires was comparable to other blended care interventions that combine face-to-face sessions with an EMI, which have reported 25% to 48% engagement during the intervention period (van Aubel et al., 2020; van Aubel et al., 2024; Rauschenberg et al., 2021; Thonon et al., 2021). Notably, we observed variability in the duration of engagement, which appeared to be influenced by the therapeutic format. Clients in individual therapy generally engaged longer, possibly due to the ongoing nature and frequency of therapy (e.g., bi-weekly sessions or longer intervals). In contrast, group-based formats typically follow a more structured format, which may have contributed to the difference in engagement. Further, clients completed exercises in the app on average five times per week (4 EMA-initiated and 1 app exercise). This finding converges with previous ACT-DL studies (Batink et al., 2016; van Aubel et al., 2020; van Aubel et al., 2024), as well as research showing higher engagement with app-initiated versus user-initiated activities (Eisner et al., 2023; Hassan et al., 2023). Regarding perceived usefulness of the ACT exercises in the app, our findings align with those from the previous ACT-DL version (van Aubel et al., 2024).

Clinicians flexibly used ACT-DL across care settings, reflecting ACT's modular and context-sensitive design. However, outpatient clinicians faced setting-specific barriers. Because outpatient sessions are typically one-on-one and client-funded, clinicians reported concerns about unpaid time to learn ACT-DL, echoing prior research suggesting that financial incentives can facilitate adoption (Weermeijer et al., 2024). This points to gaps in organizational support (e.g. resources, protected time, reward) as outlined in the Consolidate Framework of Implementation Research's inner and outer setting domains (Damschroder et al., 2022). Outpatient clinicians also worried that time spent managing the dashboard could detract from the therapeutic relationship and from valuable session time. In contrast, inpatient clinician may experience more freedom to experiment with new interventions without direct financial implications for themselves or clients. Strengthening these facilitating conditions may better support outpatient adoption.

While our findings support the feasibility of ACT-DL, there are additional considerations. First, qualitative findings linked variability in app engagement to usability issues (e.g., exercises not presented on the home screen), the limited integration of the EMA data into sessions and client-related barriers such as diagnosis (e.g., psychosis). For example, a meta-analysis on EMA compliance among individuals with severe mental health disorders found that individuals with psychosis showed significantly lower compliance compared to other diagnostic groups (Vachon et al., 2019). Variability in engagement might also reflect that the app's functionalities did not fully align with client expectations. Second, overall clinician reach was limited. On the one hand, given the timing of the study, limited reach may be explained by the COVID-19 pandemic (also see (Weermeijer et al., 2023)). On the other hand, limited reach may be explained by the complexity of EMA-based tools. Although clinicians are generally open to using EMA-based tools in their practice (Piot et al., 2022; Bos et al., 2019; Weermeijer et al., 2024), they do report some hesitancies (Ellison, 2021; Soyster et al., 2023; Frumkin et al., 2021).

### 4.3 Usability, time and competency shape integration of ACT-DL

Consistent with prior work, there was considerable variation in the use of ACT-DL (Bos et al., 2020; Weermeijer et al., 2023; Hall et al., 2025; de Thurah et al., 2025). Based on the qualitative findings, the variation in usage of ACT-DL was likely amplified by the group-session format, as EMA feedback has predominantly been examined in individual therapy contexts and mainly as assessment output rather than as part of an intervention (Bos et al., 2020; Weermeijer et al., 2023; Hall et al., 2025; de Thurah et al., 2025). Further, because outside-session support was perceived as one of the major benefits of ACT-DL, clinicians may have placed less emphasis on using the visualizations during sessions. Successful cases demonstrate that EMA was used to explore daily life experiences, evaluate treatment progress (e.g., psychological flexibility), and inform treatment decisions. These applications reflect earlier qualitative and quantitative research (Bos et al., 2020; Piot et al., 2022; Weermeijer et al., 2024) suggesting that ESM was used as intended.

Usability findings indicate that although core elements (e.g. items, visualizations, exercises) were perceived as relevant, overall system usability was limited, particularly regarding setup and interpretation. Dashboard complexity and time constraints hindered familiarization, and clinicians often needed several weeks before feeling competent. These findings echo prior work showing that clinicians struggle to translate EMA into actionable insights, and that time demands and usability impede adoption (Piot et al., 2022; Weermeijer et al., 2023; de Thurah et al., 2025; Daniëls et al., 2019). Balancing comprehensive data with clear, accessible visualizations that are both meaningful and practical to use is therefore crucial (Weermeijer et al., 2024; Schulte-Strathaus et al., 2025). A recent experimental study found that clearer textual guidance on the statistical analysis improved clinician interpretation confidence, accuracy and decision making (Piot et al., 2025). These findings emphasize the need for streamlined systems that reduce burden and offer stronger interpretative support, alongside targeted training to build clinician competency.

### 4.4 Recommendations for future research and implementation

This study provides an example of how interventions can be improved by using a User-Centered design. However, optimization is an iterative process (Kip et al., 2025) and further adaptations can improve feasibility, usability and potential for integration into routine care. Based on our findings, we have developed a list of 10 practical recommendations for future iterations of ACT-DL (Table 5). These recommendations focus on both the design and implementation of the intervention and, although framed in the context of ACT-DL, are applicable to other advanced blended care interventions that incorporate EMA and/or an EMI. Overall, in line with recent calls to action in mental health intervention research (Morton et al., 2020; Stiles-Shields et al., 2022), we emphasize User-Centered and participatory research principles.

**Table 5:** Practical recommendations for future iterations ACT-DL.

	Recommendations
1	Define the clinical function of EMA, more specifically whether EMA is used for assessment (e.g., symptom monitoring, evaluate treatment outcomes) or intervention (e.g., providing tailored exercises), but also how the data can be used, and how it contributes to therapeutic outcomes (e.g., increased self-insight, skill development through everyday practice). This can support end-users in recognizing the value of EMA in the therapy, potentially enhancing client and clinician engagement and motivation.
2	Collaborate with (ACT) clinicians to identify clinically relevant visualizations (e.g., ACT skills, affect dynamics, context, symptom monitoring) for ACT therapy. Displaying fewer visualizations on the dashboard (in both number and type) will enhance usability by balancing usefulness and practicality. Another solution may be to make the dashboard modular, so clinicians themselves can select which visualizations to present (or fix) on the dashboard, rather than presenting all visualizations.
3	Collaborate with clients to better understand their preferences and expectations of the ACT-DL app. Quantitative results showed mixed opinions regarding the app's expected features. By actively involving clients, we can gain insight into which components (e.g., EMA questionnaires, exercises) fall short of expectations and how these can be improved to better align with their preferences and needs.
4	Clearly explain the purpose of the exercises that are offered, especially with EMA-initiated exercises (e.g., ACT skill with lowest score), to help clients understand their relevance and gain insight into areas for improvement.
5	Strive for a balance between built-in flexibility and usability. While flexibility is essential for adapting interventions to different clinical contexts and individual client needs, it can also make the dashboard less intuitive to use. In the current setup, clinicians had to manually schedule each component (e.g., morning/evening questionnaires, EMA questionnaires), select items, and update ACT-specific content weekly. These demands made using the dashboard more complex and time-consuming. Collaborating with end users and User Experience designers can help identify which customization options are truly valuable and which introduce unnecessary complexity. Enhancing the usability of the dashboard, by streamlining navigation and making adjustments more intuitive, could reduce clinician burden and support sustained use.
6	Develop visualizations or features tailored for using ACT-DL in group sessions. Half of the clinicians in our sample delivered ACT-DL in a group format. One clinician who integrated EMA data into group sessions created paper-based overviews herself to facilitate discussion. Introducing group-level visualizations or features that allow for easy printing could support broader integration of the data into group settings, without requiring separate sessions solely for data discussion.
7	Enhance usability by piloting the dashboard and app with end users before implementation. This can involve collecting feedback through surveys or interviews, as well as using Think Aloud procedures where, for example, clinicians are asked to verbalize how they would describe certain visualizations (Carter-Roberts et al., 2021). These methods can reveal usability challenges (e.g., interface, adaptability, type of visualizations used) and but also potential implementation barriers.
8	Develop pragmatic training materials (e.g., short manual, video tutorial, cheat sheet, online website) that are directly applicable to the daily tasks of clinicians, keeping in mind their limited time. The materials should include step-by-step instructions, preferably in various formats (e.g., text, video) to accommodate different learning preferences. In addition, training materials can be integrated into the system, allowing clinicians to easily access them when needed to support their use of the dashboard
9	Offer clinicians practical hands-on training on using, interpreting, and discussing EMA data, including basic dashboard functions, navigation, and advanced features, but also on how to address challenges with client engagement (e.g., difficulties complying with questionnaires, exploring reasons for missing data). In this study, training focused primarily on technical aspects, which may have limited clinicians' understanding of how to integrate EMA data into therapeutic conversations. Interactive workshops with use-case examples, step-by-step guidance, and role play can build skills and competence.
10	Tailor implementation strategies to each healthcare setting (e.g., inpatient, outpatient, individual or group session), to improve integration in current workflow. For example, use-case examples can illustrate how to collectively discuss visualizations in group settings. In outpatient care, strategies might focus on discussing EMA data briefly, highlighting which visualizations are most crucial to discuss in the session. Although guidance on which visualizations to discuss during each session was included in the manual used in the current study, embedding this information in a hands-on training can make it more accessible and increase uptake.

Beyond practical recommendations, it is crucial to define meaningful engagement within the context of the intervention (Cipriani et al., 2025). Clear definitions will help researchers and clinicians to interpret engagement patterns and clarify clinical purpose, which may vary between assessment and intervention. Future research should examine how engagement relates to outcomes (Cipriani et al., 2025) and explore client, clinician and contextual factors that influence use and effectiveness of interventions like ACT-DL.

### 4.5 Strengths and limitations

This is the first study demonstrating a complete iteration of ACT-DL, describing its optimization and evaluation in clinical care. However, several limitations should be noted. First, the small, homogenous sample of women psychologists limits generalizability to other professionals (e.g., psychiatrists, other health workers) and gender. Second, the dashboard lacked a pause or delete function, causing questionnaires to continue even after client dropout, making it unclear whether non-responsiveness reflected disengagement or scheduling issues. Third, the absence of client interviews limits insight into user experiences, which are vital for meaningful refinement.

## 5 Conclusion

This study provides valuable insights into the optimization, usability and implementation feasibility of a blended care ACT intervention, ACT-DL, in clinical care. It demonstrates a user-centered approach in developing and refining complex digital interventions. Our findings contribute to the growing evidence supporting the feasibility and usability of ACT-DL. Clinicians employed various strategies to incorporate ACT-DL but encountered several barriers relating to lack of time, usability and client characteristics. While competencies improved over time, challenges remained in mastering and integrating visualizations into practice. These insights are essential for optimizing future blended care interventions and ensuring their successful integration into existing healthcare practices.

## Abbreviations

ACT: Acceptance and Commitment Therapy
ACT-DL: Acceptance and Commitment Therapy in Daily Life
EMA: Ecological Momentary Assessment
EMI: Ecological Momentary Intervention

## CRediT authorship contribution statement

EVA, MW and IMG were involved in designing the study and setting up the data collection procedures. LU, EVA and MW were involved in data collection procedures, manuscript writing and data analysis, during which LU received feedback from JRB, JW and IMG. Moreover, all authors contributed to the review of the manuscript and approved final version.

## Declaration of competing interest

None declared.

## Data Availability

The data is available upon reasonable request through the Data curation for OPen Science (DROPS) system in REDCap (Harris et al., 2009), hosted at KU Leuven. Researchers can submit an abstract, later reviewed by the research team to ensure research is not overlapping. After the abstract is approved, the researcher preregisters the analysis plan and the dataset and variables of interest will be provided by a data manager.
