# Supplementary material for: Refining acceptance and commitment therapy in daily life (ACT-DL) for clinical care: Optimization and mixed-methods evaluation of usability and implementation feasibility
Source: Internet Interv. 2026 Jul 22;45:100979. doi: 10.1016/j.invent.2026.100979 (PMC13450586; doi:10.1016/j.invent.2026.100979)
Supplement: Supplementary file 1 [file mmc1.docx]

# **Supplementary Materials for ‘Refining ACT-DL for Clinical Care: Optimization and Mixed-methods Evaluation of Usability and Implementation Feasibility**

1. **Quantitative Analyses**
   1.1. **Additional Tables**

**Table S1**

*Sociodemographic Characteristics for Clinicians and Clients*

| **Characteristics** | **Participants using ACT-DL** |
| --- | --- |
| **Clinicians** | 7 |
| Age (years), mean (SD) | 39.14 (11.46) |
| Sex (female), n (%) | 7 (100%) |
| Profession  Psychologist, n (%)  Years of ACT therapy, mean (SD)^a^ | 7 (100%)  5.86 (4.88) |
| **Clients** | 30 |
| Age (years), mean (SD) | 32.47 (13.56) |
| Sex (female), n (%)  Diagnosis (yes), n(%)  Psychotic disorders  Mood disorders  Developmental disorders  Post Traumatic Stress Disorder  Burn-out  Borderline Personality Disorder  Medication use (yes), n (%)  Prior experiences with therapy (yes), n (%)  Education (high education), n (%)  Employment status  Full time, n (%)  Part time, n (%)  Unemployed, n (%)  Student, n (%)  Self-employed, n (%)  Retired, n (%)  Social benefits, n (%) | 13 (43.33%)  17 (56.67%)  4  7  6  2  1  1  20 (66.67%)  20 (66.67%)  14 (46.67%)  6 (20%)  2 (6.67%)  6 (20%)  3 (10%)  1 (3.33%)  2 (6.67%)  10 (33.33%) |

^a^ Given that years of ACT therapy was based on open-text responses, estimates are approximate.

**Table S2**

*Individual Item Scores for Clinician Usability Questionnaire*

| **Item** | **Mean (SD)** |
| --- | --- |
| It was easy for me to learn to use the dashboard | 4.57 (0.98) |
| Overall, I found the ACT-DL dashboard easy to use | 4.43 (0.98) |
| I thought the dashboard looked nice | 4.26 (0.76) |
| The design of the dashboard made it possible to use all functions easily, such as adding a client | 5.86 (0.69) |
| The design of the dashboard made it possible to use all functions easily, such as making a questionnaire | 4.86 (0.90) |
| The design of the dashboard made it possible to use all functions easily, such as planning questionnaires | 5 (2.08) |
| The design of the dashboard made it possible to use all functions easily, such as creating sessions | 5 (1.41) |
| The design of the dashboard made it possible to use all functions easily, such as assigning sessions | 5.14 (1.57) |
| The design of the dashboard made it possible to use all functions easily, such as visualizing data | 3.86 (1.68) |
| The dashboard had all the features I expected | 4.14 (1.77) |
| I found the exercises in the ACT-DL app relevant | 5.71 (1.38) |
| I found the items in the questionnaires relevant | 5.14 (0.38) |
| I found the visualizations on the dashboard an added value to the therapeutic process | 4.28 (1.98) |
| I found the visualizations relevant | 5.14 (1.07) |
| I thought the app and dashboard added value to the therapeutic process | 4.71 (1.89) |
| I thought I spent too much time on the dashboard (R) | 4 (1.63) |
| I would use the dashboard again | 4.14 (1.35) |
| Overall, I was satisfied with the dashboard | 4.43 (1.51) |
| It would be useful for me to continue using the dashboard | 4.57 (1.51) |
| Total Usability score | 89.29 (16.85) |

*Note.* The total usability was calculated using the sum of all items, resulting in a maximum usability score of 133.

**Table S3**

*Individual Item Scores for Client Usability Questionnaire*

| **Item** | **Mean (SD)** |
| --- | --- |
| From the start, I found the app easy to use | 5.3 (1.63) |
| In general, I found the app easy to use | 5.3 (1.69) |
| I liked the design of the ACT-DL app | 4.45 (1.79) |
| The information on the ACT-DL app was well organized, I could easily find what I needed | 4.65 (1.60) |
| When I made a mistake on the ACT-DL app, I could correct it easily and quickly | 4.1 (1.45) |
| The design of the ACT-DL app made it easy to use all the functions, such as creating exercises, adding a practitioner, adjusting the settings | 4.55 (1.50) |
| The ACT-DL app had all the functions and features I expected from it | 4.05 (1.93) |
| I understood the information about the ACT skills well | 5.2 (1.67) |
| I understood the tasks in the exercises well | 4.95 (1.67) |
| I understood the items in the questionnaires well | 5.15 (1.60) |
| I felt comfortable using the ACT-DL app when I was with other people | 5.35 (1.64) |
| I spent too much time on the ACT-DL app (R) | 3.9 (1.45) |
| I would use the ACT-DL app again | 4.5 (2.14) |
| I was confident that all the information I entered on the ACT-DL app was received by my clinician | 5.35 (1.57) |
| I felt comfortable talking to my clinician about the data collected in the ACT-DL app | 4.2 (1.46) |
| The graphs of my data were clear | 4.5 (1.61) |
| The ACT-DL helped me to practice the exercises/metaphors in daily life | 4.5 (2.01) |
| Through using the ACT-DL app, I became more aware of my feelings and thoughts | 5.05 (1.57) |
| The ACT-DL app made me more aware of how I deal with difficult feelings and thoughts | 4.65 (1.53) |
| Through the ACT-DL app, I better understand what my values and goals in life are | 4.4 (1.35) |
| The ACT-DL app helped me understand how to deal with difficult feelings and thoughts | 4.3 (1.45) |
| Total Usability score | 98.4 (23.13) |

*Note.* The total usability was calculated using the sum of all items, resulting in a maximum usability score of 147.

**Table S4**

*Distribution of reported technical and practical problems and examples*

| **Reported problems** | **Frequency** | **Example** |
| --- | --- | --- |
| **Clinicians** |  |  |
| Receiving data | 6 | Platform down, insufficient questionnaires scheduled |
| Visualizing data | 3 | Incorrect graphs due to difficulties in receiving data |
| Setting up questionnaires | 3 | Difficulties installing ACT-DL applet |
| Planning questionnaires | 2 | Beeps scheduled for too long – system ran very slowly |
| Assigning sessions in the app | 0 | N/A |
|  |  |  |
| **Clients** |  |  |
| Loading applet | 1 | N/A |
| Text in exercises was not readable | 4 | N/A |
| Problem with images in the applet | 8 | N/A |
| Problem with audio in the applet | 40 | N/A |
| Problem with video in the applet | 0 | N/A |
| Problem with notifications | 8 | N/A |
| Others ^a^ | 56 | N/A |
| *Error message in evening questionnaire* | 34 | N/A |
| *Notifications did not work* | 3 | N/A |
| *Repeating questions* | 1 | N/A |
| *Limited textbox in applet exercise* | 1 | N/A |
| *App freeze* | 1 | N/A |

*Note.* N/A = not applicable.

^a^ Based on open-text answers provided by clients.

1.2. **Additional Analyses: Perceived Competency**

Given the small sample size and data points per clinician, the visual exploration of changes in perceived competencies is exploratory and should be interpreted with caution. For constructing (Figure S1) and planning (Figure S2) the questionnaires, the graphs demonstrate variability in the initial competencies at the start of the intervention. Clinician 1 and 6, both working in outpatient care, showed lower levels of perceived competencies and greater variability over time, compared to other clinicians. When looking at the open-text answers, clinician 6 reported difficulties with scheduling questionnaires when clients particularly have irregular schedules “*I have not yet mastered the dashboard. It becomes especially puzzling and figuring out with clients who cannot have a standard notification schedule; then a lot of actions have to be done and that takes a lot of time and some opportunities to make mistakes.",* and later, when the competency at the graph dropped at week 9, she explained the following: “*Been a while since I had to change the notification schedule again and was back searching a bit for exactly how to do this. Was some fumbling, also due to having little time”,* and was reported again at week 16.

For visualizing (Figure S3) and explaining (Figure S4) the data, the clinicians show a similar pattern; all report generally low to no competencies at the start of the intervention period. Over time, perceived competencies in visualizing and explaining the data increase, with approximately four weeks until clinicians report positive competencies. After reaching positive competency, the levels seem to remain stable. Exploring the open-text items indicate prior difficulties with the high amount of data that clinicians receive on the dashboard.

*“I am not yet comfortable using the data, in the preparation I also found it not so easy to look at the visualizations and extract useful info for the client”* (T1) – week 1

“*With charts, visualizations there are SO MANY choices, it is quite time intensive to determine this at the group level what might be of interest to a group. Moreover, there are no standard printouts to be found of all visualizations so you have to manually select to organize session*” (T2) – week 3

“*Explaining the data is sometimes confusing when it comes to the evolution on the different skills. Do high scores represent more competence or just less (answer that appears when your cursor hovers on it is rather confusing given that this seems to insinuate that it is actually more burden and less competence)?"* (T1) – week 9

| **Figure S1**  *Temporal Variability of Constructing Questionnaires* | **Figure S2**  *Temporal Variability of Planning Questionnaires* |
| --- | --- |
|  |  |

| **Figure S3**  *Temporal Variability of Visualizing the Data* | Figure S4  *Temporal Variability of Explaining the Data* |
| --- | --- |
|  |  |

2. **Qualitative Analysis**

**Table S5**

*Theme and example quote for deciding to use ACT-DL*

| **Sub-theme** | **Clinician** | | **Quote** |
| --- | --- | --- | --- |
| Evidence-based methods | T1 | And then, of course, my quest from my clinical practice to always continue working in an evidence-based manner, but also to incorporate that blended element. You can't always offer it yourself, not for free. So I thought, I really want to figure this out. And then I thought, Well, that's a good opportunity. | |
| Support in between sessions with centralized place for exercises | T5 | So the idea that between sessions, because we do four sessions a week, a patient who has such difficulty retaining things could benefit from repetition. And I had been looking for a way to do that for a while, wondering how they could take things with them outside of the sessions. And that was with printed homework assignments. But they just left them lying around. Well, yes. And the idea that it's actually nice that there is.. not so much that I get the input, but that it's a reminder for them. | |
|  | T6 | And secondly, um, in my therapy, I was and still am very focused on how I can best support people in implementing the things we look at and practice in therapy at home or outside of the sessions. And I noticed that this is not so obvious for clients. For example, clients say, "It worked in the session and you were there, but when I have to do it on my own, it's difficult or sometimes I'm less motivated." So that's something I was working on, and when I heard about this app that actually works around those things and also tries to implement things in everyday life, I thought, "*Oh, this could be a solution*." | |

**Table S6**

*Themes and examples for ACT sessions*

| **Sub-theme** | **Code** | **Clinician** | **Quote** |
| --- | --- | --- | --- |
| Flexibility |  | T1 | And that I've become a little more gentle in how I go about it [the intervention protocol]. *Look, if it's not there, okay, it's not there, okay.* Whereas with my first client, okay, that has to be good and everything has to be in it. So, I have to follow that so strictly. So I think I can shake that off a little bit more as I've started.  I did follow it pretty well. Toward the end I didn't follow everything that was in the protocol. Sometimes I didn't go through things, but I did follow all these different days like that and tried to keep that order, though. But here and there I guess that was interspersed with pieces of how I do it differently, but actually followed the order pretty well. |
|  |  | T2 | Wait, hey, we got the sequence, I think, I do think the sequence was similar because *what do we always do?* We do acceptance, diffusion, I think then contact, then self as context, then value and committed action, so I don't see any, I do think in terms of sequence it was similar. Now, there was also in the protocol something about psychological flexibility and creative hopelessness which we don't have, we don't have those separately in the session, so I didn't put those in there either because that's something that is discussed in all the different sessions. |
| Group format | Dynamics | T2 | They could indicate this [if they wanted to discontinue the app] honestly, yes, but then it was also the group aspect, which I think was difficult because you have people who want to stop and then you have to be careful that they don't contaminate the whole group with that demotivational element.  Yes, I always handed them out individually because I thought, yes, here in a group, that might be a bit challenging, but I always said, um, if anything was unclear or if they wanted to discuss it in a group, I had the computer with me, so I could have projected it, but no one ever asked for that. It was more like, "Oh, maybe, that's how it was for me and, oh, wow, you have so much variety here, I have so little,“ so it was also mutual curiosity and then asking questions like, ”Oh, why am I going up and down so much?" So that too, it had something, yes, it stimulated curiosity and that in itself is already good, right? Yes, because in the end, you see them in session, yes, you have quiet people who don't talk much, yes you don't know about that either, of that is difficult, that is difficult. Yes, and I know about the loudest ones, "I can't accept or I miss that", but you don't know about the quieter ones and I found it very interesting to have some insight [trough the visualizations] into that. |
|  |  | T8 | I've been thinking about that, because in the end they filled in the app and you don't do much with it. Uhm, it would make a difference if more people in the group filled it in. Then you would be so-because now only two are the only ones who were working on it. I do think that with [client 8.01] she also picked up things separately, but in group where everyone was there, I didn't do anything with that [the app]. |
|  | Structure | T2 | So then it was actually one topic every week and then during the first session on Tuesday we did the feedback from the week before … and that they could first talk in a group about how it went, what they encountered, then look at the results and see if they came across anything striking or that they didn't understand or that they wanted to share. Yes, and then try to come to a group decision on that |
|  |  | T5 | Far too little used it myself to get anything out of it. So, discussing that then so in between actually happened very little while that was the idea beforehand. We will discuss it in individual conversations, but yes, we will. |
| Individual format | Balancing app use in therapeutic alliance | T1 | I do think you have to be extra vigilant that you don't become too task-oriented. Uhm, I think you have to keep paying a lot of attention, also to the therapeutic relationship so that you don't lose it. I think you should focus more on it. Uhm. yes. And that that's what happens. If you are alert to it, and you look carefully at which way and then not too long with that dashboard, but also enough time for the here and now. Making sure that people can still contribute their experiences, but also that you can still add your content. That complexity I found, uhm, yes, you had to be very attentive to. |
|  |  | T6 | What I noticed is that I tried to follow the flow of the ACT protocol, but there were also regular sessions where the client actually came in and wanted to discuss something that didn't really fit in with that protocol. So then I had to let go of that [the intervention protocol] at times and we actually did and had to do something else. And that in itself isn't really a problem, but yes, it does of course give the app a different place in the session.  And I sometimes did it [using the dashboard] with the client in the session, especially in the beginning. But then I found- *ow, this is a waste of time for the client. Who pays for his session*-so if I didn't find it immediately, I left it and then that became a to-do for me at the end of the day or for the next day, but you can't leave that too long either, because the person asked for other notifications. Otherwise, that person will keep getting the old notifications. And then, that eventually became a to-do for the end of the working day to sort it out then. |
|  | Financial aspects | T6 | Because I could stretch a whole day or several days to do that [familiarizing with ACT-DL] more thoroughly, but appointments are scheduled. And indeed you can perfectly free up space, but then you avoid that as much as possible. And then that also has to do with the financial aspect. That you have no income then, so yes.  And I did it especially in the beginning sometimes with the client in the session. But then I found-*oh, this is a waste of time for the client.* Who is paying for his session- |
|  |  | T7 | That the client knows why they want to participate in it and why that, and you can tell that in therapy, but at that point your client pays for it and they say "You're actually recruiting me here", and then during a therapy session. That's not cheap for a client. So I also try to give as much as possible to a client, during a session. And if that client feels like *there's a lot of time being put into an app that doesn't contribute anything for me, then I'm paying a lot here,* of course. |

**Table S7**

*Themes and example quotes for ACT-DL EMI*

| **Sub-theme** | **Code** | **Clinician** | **Quote** |
| --- | --- | --- | --- |
| Personalization | Yes | T1 | No, the only thing I had to adjust once is that someone said to me "But in that context that I don't understand well or - " Yes, I think that was [client 1.03], who is very quickly anxious in the supermarket or public transport, for example. And we then added that to that, for example. That he said of "Yes, I want to indicate that, but it's all in 'other' now and I can't do that." But other than that, I really didn't have any, yes, that or that |
|  |  | T2 | Right, yes, we matched the beep schedule to the therapy schedule |
|  | No | T2 | No, I actually just left everything as it was, I found it standard already time-intensive enough that I thought if I had to start personalizing [items] again now, no, that was on a group level I don't think, not ideal. |
|  |  | T7 | For now, I haven't. I have no additional questions-because, his response was already "It was already seven times." So, if I then ask again- that he would get completely overwhelmed. |
| Clinician as facilitator | App engagement | T4 | So in terms of those visualizations, I tried to be motivational and showed to the clients of "You can get all this out, you had to fill it in." |
|  |  | T6 | Uhm, but I think for me it was also- uhm. searching to keep that balance of how can I still motivate her somewhat to do that, but without her feeling pushed? I didn't find that so obvious. … Because, I really did validate that. I understand that it is not obvious to get a notification so many times a day. So, then she must have felt understood about that. In the beginning I did try to encourage her a bit, so gently. But I felt it didn't work and I let it go. |
|  | Compliance and coping | T5 | So, there really have been regular conversations about that [the beeps] too. Yes, that has been a theme. So that app and those beeps. Whether they find it stressful and whether or not they get filled in. Or how confrontational - so in that sense it did bring a lot without me bringing up those graphs. |
|  |  | T6 | So that was something that we would then work on within the session on and on, but that's an example of yes-something that might seem simple: Just open your mobile phone and fill that in, but that's actually not that simple for a lot of clients. Because there are some like [client 6.01] who develop like that anyway, of, yeah*, oh no that notification again.* And then that really became a huge barrier that she didn't get over. And she herself didn't want to push it either, saying it's important after all. We always discussed it, but it's a difficult one. |
|  | Clarifying app content |  | for example those, those things that arrowed towards the 6 ACT skills so, I know there were so a few who said for example of yes, the self as context, that that was very bizarrely asked and that they actually didn't quite know, yes, what do they mean by that? |
| Discussion of ACT-DL EMI | App exercises | T5 | So then we discussed that every week. Just like we discuss the homework assignments back every week. “How did the exercise go. We did the same with the applet: How did the exercise go? Which one did you choose? Which one suits you best? Did you do the listening exercise?” Ah, you did the exercise with the cartoon. And you have- uhm, so that has indeed been brought up again every week. |
|  | No use of visualization | T5 | Yes, because you don't want to lie around and say that? That you then felt really bad on Saturday. And if the patient then says, “no, Saturday was great, a family party, I haven't been this happy in a long time-“, ah, yes, then the scale is reversed. Then that does indeed mean that Saturday was really good- but that's also my insecurity, you know. That I had less control in that and if I had just jumped and tried, it might have paid off. But I feel- I am not sure for myself and I don't want to make a fool of myself either, which is a lot, but so in that vein. But, I also don't want them to lose faith in the app or anything. So, yes, they have to fill it in but she then said things that are not true or she herself didn't know exactly what she was saying. |
|  |  | T8 | Uhm, it is a group therapy, so I have not been able to relate it back to uhm the persons I have seen in the group. The other one who dropped out, I did, I did say something to her separately. But there is no room for that in the group. Actually that is a tight program and I don't have extra space for that. |
|  | Use of visualization | T1 | And for example with another girl who is very extroverted, and I think very often in sessions, she shows a lot of emotions and because she's having a conversation, she cries faster and so on. That I could also see, ah yes, it's actually okay with you. So, there are a lot of cool moments like that. … And through those sessions I was able to do that. And then you also see that that's valuable to have that in there. I think with that one client I was able to wrap up because of the protocol. Did I really stop that therapy now, hey. Whereas otherwise I might have been so anyway maybe one more time like "Ah yes, maybe I still need it." Whereas now I could say like "Look, one time here." |
|  |  | T2 | For example, what you then picked up from someone who has been withdrawing a bit more lately and who is shorter and more compact, and then suddenly hey, in the beginning I didn't do that as standard, but then I pulled out that sleep chart and it turned out that that person also, yes, subjectively experienced that she hadn't slept well on a single night that week, yes, then that also puts that into context. Then next time you ask, 'Ten, I've seen that, does that count? And then she will come to say that too, while yes, that does help, to find out that she is reporting a lot of negative affect and we see that too, she is withdrawing a bit more, she is sitting in her room a bit more, but how does that come about and then it will come out, part of it is also that she had nightmares and then she could talk about nightmares. |
|  |  | T6 | But what I thought was a really interesting one, what I tried to do at the end, is that flexibility. Look, you've gone through all that protocol now, let's see. What have you grown in and what maybe not? |

**Table S8**

*Themes and example quotes for barriers to integration*

| **Code** | **Clinician** | **Quote** |
| --- | --- | --- |
| Time | T5 | I also find that very difficult to then block out time to read something that potentially contributes something, but potentially may not. Depending on how well they fill it in or how much they dwell on it. So the thinking has often been to just look in the manual to see if there is info in there. And I just haven't done that. |
|  | T6 | Again, that's time- then I am more likely to make the choice to go through my notes from last time, rather than throwing the system open and having to spend some time figuring out how to make the right visualizations. I always have 15 minutes between each session. Sometimes talks also run out. So there is actually not much time. I also can't always look at my notes from the previous sessions. |
| Clinical approach | T5 | I also ended up not starting ambulatory because that's towards just ACT. So then we go into those processes like grief counselling, and all-that's much more intertwined. That's much less nicely delineated. |
|  | T6 | But, it also has something contradictory for a bit, with ACT. In the sense of, that is recorded, uhm-which positive feelings, negative feelings and complaints. And that's something that within ACT you just don't focus on, because that can then create the illusion of, you can do anything. And I think that's also a bit, on a theoretical level then, wrong. In the sense of, I don't see any added value in focusing on that in therapy, so we're not going to do those visualizations. |
|  | T7 | … but the way it came across to the client, it was actually- it actually went against my approach because it was actually quite rigid, I thought, of feeling and looking at you at certain times. Whereas that's what I'm actually trying to get that flexibility into it. That people feel more, yes- from the moment they feel changes in their body, that they start looking at what they need. |
|  | T2 | So we normally have 2 theoretical sessions of 1h actually, and then Thursday 1 practical session, again of 1h but we then during the study chose to retain 1 of those 2 sessions actually yes, to actually really go with the app, to be able to do the feedback at group level. So then it was actually one topic every week and then during the first session on Tuesday we did the feedback from the week before. |
| Mastery | T4 | Um, first, um, I installed it on my cell phone and saw how it worked. And then I did a few fake test subjects, not clients, just to see how it all works and how I can install and visualize it flexibly. ... That way, I also wanted to get enough practice on clients who are actually volunteers, who aren't really patients. Uh, because once you understand how to use it, it's not complex. |
|  | T7 | Yes, I think also a good education. That also as a therapist you know how it all works and what that is behind it, because then you can maybe also start to put more emphases of "This is important for you." And the other person might be that. And then you can start to individualize it a bit more, and I think that can also be important. |
| Usability | T2 | Yes, we went to another building where there is wifi, and then I was told by the reception desk "you're not allowed to come here with patients for the wifi", so that was actually a bit of a false start, but then in the end everyone installed it there, but afterwards it turned out that I had added an incorrect researcher or something, or at least I had added something wrong so that we didn't get to see everything properly, so in the beginning, that was also a bit of a hassle and that was the group aspect as well, if there is no wifi here, yes, otherwise if you can just quickly do that here on the wifi after your session, yes then I don't think it's so much work but that was an extra step of going there with the group |
|  | T4 | About the visualizations. I still found that scheduling relatively easy, but those visualizations, uhm, yes-and especially then when there was so much difference. Some people filled it in, others didn't. Then I thought Is it me that I can't see it? Or is it because there is no data? I sat on that a bit. |
|  | T5 | I think-that's such an amount of info with lots of things you can tick and un-tick. And that was then initially all ticked so it was so one graph where I thought I don't know what I'm seeing here in colors. Because you can move your mouse over it and it tells you something but you don't know what. |
| Type of client | T2 | I also noticed that for some people it was too much. They couldn't manage that well themselves, like, okay, now I'm going to skip a beep, no, then that was immediately gate closed and, and really not allow anything more in there. While others, yes, the somewhat, more strongly structured people, I think, who were happy with that and who, yes, they were also curious, especially those first weeks, wondering what is this, what is coming out of this [the visualizations]. … They were then given that [printout of visualizations], and some of them said, "Could we come back to that individually?" for example. |
|  | T4 | … What I mostly heard when I asked of "How come you fill it in so limited?" Then it was like "That they get so many beeps because they have so much on, and then they are quite inclined not to respond." And to then also be busy with other beeps from social media rather than that from IMPACT. And also very often not having their mobile phone with them in the room. Not having it charged. And that's another thing, the disorganized nature of psychosis. |
|  | T1 | I think with my first client, I followed the protocol as it was presented most closely. Now, that was also a client who lent himself to that. He had a number of primary complaints. Not too much comorbidity. So that was really someone where it went according to the textbook... But that's how it went. And I also had the feeling that the structure allowed for that. But, for example, with [client 1.01], where there was more complexity, I did have to switch things around a bit. And that was really necessary. And I did discuss some things and not discuss others. |
|  | T5 | Yes, but I do feel that those who were able to motivate themselves to actually use it in between sessions were also the ones who were motivated about the program itself. They benefited greatly from it. And those who were not required to follow the group program and did not see much need for it themselves used the app less. And so they felt less value in it, I think. |
